# The association between diabetes mellitus and reduction in myocardial glucose uptake: a population-based ^18^F-FDG PET/CT study

**DOI:** 10.1186/s12872-018-0943-9

**Published:** 2018-10-29

**Authors:** Lijun Hu, Chun Qiu, Xiaosong Wang, Mei Xu, Xiaoliang Shao, Yuetao Wang

**Affiliations:** 1 Department of Radiation Oncology, The Second People’s Hospital of Changzhou, Nanjing Medical University, Changzhou, 213003 Jiangsu China; 2 Department of Nuclear Medicine, The Third Affiliated Hospital of Soochow University, Changzhou, 213003 Jiangsu China

**Keywords:** Diabetes mellitus, FDG, PET, Insulin resistance, Nonalcoholic fatty liver disease

## Abstract

**Background:**

In diabetes, dysregulated substrate utilization and energy metabolism of myocardium can lead to heart failure. To examine the dynamic changes of myocardium, most of the previous studies conducted dynamic myocardial PET imaging following euglycemic-hyperinsulinemic clamp, which involves complicated procedures. In comparison, the whole-body ^18^F-FDG PET/CT scan is a simple and widely used method. Therefore, we hope to use this method to observe abnormal myocardial glucose metabolism in diabetes and determine the influencing factors.

**Methods:**

We retrospectively analyzed PET/CT images of 191 subjects from our medical examination center. The levels of FDG uptake in myocardium were visually divided into 4 grades (Grade 0–3, from low to high). The differences in clinical and metabolic parameters among diabetes mellitus (DM), impaired fasting glucose (IFG), and normal fasting glucose (NFG) groups were analyzed, as well as their associations with myocardial FDG uptake.

**Results:**

Compared with NFG and IFG groups, DM group had more cardiovascular-related risk factors. The degree of myocardial FDG uptake was significantly decreased in DM group; when myocardial FDG uptake ≤ Grade 1, the sensitivity of DM prediction was 84.0%, and the specificity was 58.4%. Univariate analysis showed that the myocardial FDG uptake was weakly and negatively correlated with multiple metabolic-related parameters (*r* = − 0.173~ − 0.365, *P* < 0.05). Multivariate logistic regression analysis showed that gender (male), HOMA-IR and nonalcoholic fatty liver disease (NAFLD) were independent risk factors for poor myocardial FDG uptake.

**Conclusions:**

Diabetes is associated with decreased myocardial glucose metabolism, which is mediated by multiple metabolic abnormalities.

## Background

Free fatty acids and glucose are the major substrates for myocardium energy metabolism. The process of substrate selection is dynamic and largely depends on substrate availability, oxygen concentration, and myocardial workload [1]. Under pathological conditions (such as diabetes, myocardial ischemia, and left ventricular hypertrophy), the abnormal substrate utilization, energy metabolism disorder, mitochondria dysfunction, and ATP synthesis failure [2], can all lead to myocardial structural changes and reduction in systolic/diastolic function, which then trigger heart failure. Therefore, there is an increasing demand for accurate and non-invasive imaging approaches to observe various aspects of myocardial substrate metabolism.

Diabetes is a metabolic disorder characterized by hyperglycemia, dyslipidemia and insulin resistance [3]. Diabetic patients are at higher risks of having coronary artery disease (CAD) and heart failure, exhibiting relatively high prognostic impact and a 2.5-fold increased incidence of heart failure compared to general population [4, 5], as well as an 8% increase in the risk of getting heart failure when glycosylated hemoglobin (HbA1c) is increased by 1% [6]. Studies have demonstrated that ^18^F-FDG PET/CT imaging was able to show glucose metabolism in various organs/tissues in diabetic patients, mainly because that the levels of myocardial FDG metabolism were closely related to the expression of glucose transmembrane transporter (GLUT-4) [7] and the changes in left ventricular functions [8]. However, all the imaging processes in previous studies used dynamic PET scan after the standardization of myocardial substrates metabolism environment (euglycemic-hyperinsulinemic clamp). And thereby the imaging procedure was complicated and time-consuming [9]. Currently, the most widely used imaging technique in clinic is the fasting static whole-body PET/CT scan, which is mainly used for diagnosing and staging malignancy, and also has an important role in evaluating ischemic heart disease. Although the non-specific ^18^F-FDG uptake of myocardium in whole-body ^18^F-FDG PET/CT is a common problem and can affect the result, the study from Lee YH et al. [10] showed that the myocardial FDG uptake was significantly correlated with cardiac diastolic function. Therefore, this method may have potential advantages in observing myocardial glucose metabolism in diabetic patients. In this study, we reviewed the fasting static whole-body PET/CT images of 191 subjects from our medical examination center, and analyzed the differences in myocardial FDG uptake among diabetic (DM) patients, impaired fasting glucose (IFG) group, and normal fasting glucose (NFG) group, in order to explore the clinical and metabolic factors affecting myocardial FDG uptake. Also, we explored the feasibility of using static whole-body PET/CT as a potential monitoring and follow-up tool for diabetic cardiomyopathy.

## Methods

### Study subject

**Fig. 1:**
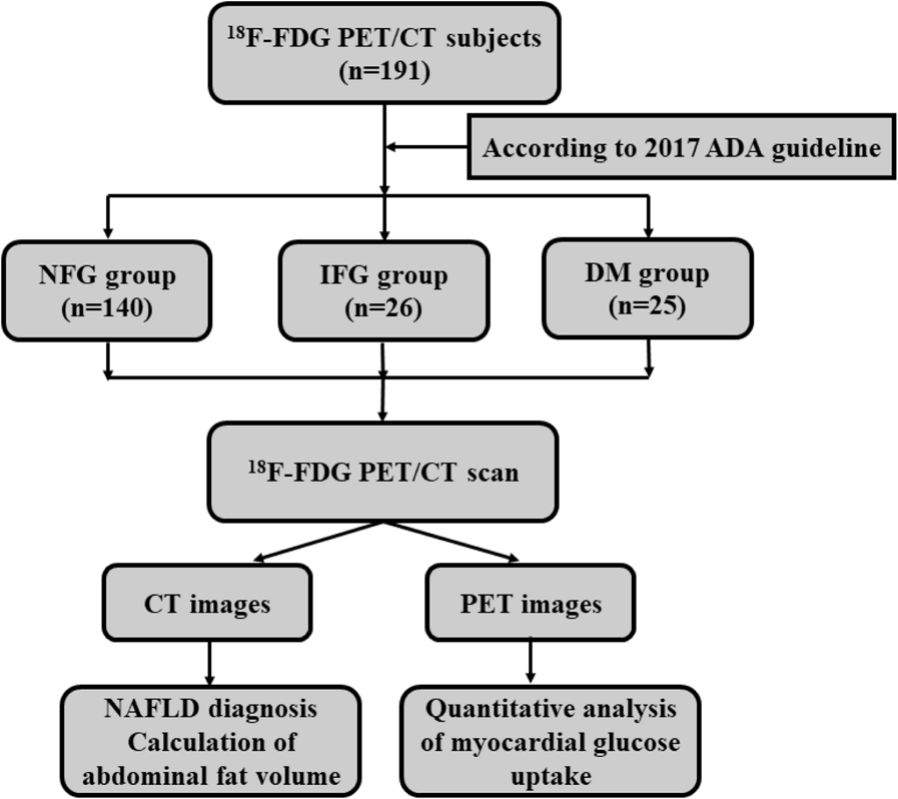
The flowcharts of subjects grouping, PET/CT scanning and image analysis. ADA: American Diabetes Association, NFG: normal fasting glucose, IFG: impaired fasting glucose, DM: diabetes mellitus, NAFLD: nonalcoholic fatty liver disease

We did retrospective analysis on the subjects who required ^18^F-FDG PET/CT examination at the medical examination center of our hospital from October 2010 to December 2015. Subjects with the following diseases were excluded from our study: acute liver/kidney dysfunction, coronary heart disease or heart failure within the past 6 months, history of myocardial infarction, dilated/hypertrophic cardiomyopathy, history of malignancy, insulin-dependent diabetes, oral administration of steroid hormones/biologics, vasculitis, collagen diseases, acute and chronic infections. A total of 191 subjects were enrolled in this study. Their gender, age, height, weight, body mass index (BMI), smoking habit (excluding passive smoking) and drinking habit (at least once a week) were recorded. The flowchart of our study protocol is shown in Fig. 1. The study protocol conformed to the tenets of Declaration of Helsinki and was approved by the ethics committee of the third affiliated hospital of Soochow university.

### Measurement of laboratory parameters

The blood samples of the subjects (fasting venous blood 5 mL, subjects were fasted for at least 8 h) were collected before ^18^F-FDG PET/CT scan. Enzymatic methods were used to measure liver functions (Alanine transaminase (ALT), glutamic oxalacetic transaminase (AST), glutamyltranspeptidase (γ-GT)), blood lipid (cholesterol (TC), triglyceride (TG), high density lipoprotein cholesterol (HDL-C), low density lipoprotein cholesterol (LDL-C), Lipoprotein (a)), and fasting blood glucose. The instrument we used was Hitachi 7600–120 automatic biochemical analyzer. The electrochemiluminescence method was used to determine the levels of thyroid stimulating hormone (TSH) and fasting insulin, conducted by Roche Cobas 8000 automatic electrochemical immunoanalyzer. High pressure liquid chromatography was used to measure fasting glycosylated hemoglobin (HbA1c), performed on BIO-RAD D-10 HbA1c meter. According to the criteria for medical diagnosis and treatment of diabetes, which were formulated by American Diabetes Association in 2017 [11], the patients with fasting glucose ≥7.0 mmol/L, or HbA1c ≥ 6.5%, or previously diagnosed as diabetes were all classified as DM group; the patients with fasting blood glucose ranging between 6.1 mmol/L and 7.0 mmol/L were classified as IFG group; others were classified as NFG group.

### PET/CT scan

The imaging machine was the Biograph mCT (64) PET/CT scan machine from German Siemens, and ^18^F-FDG (radiochemical purity > 95%) was used as imaging agent. The subjects were fasted for more than 8 h before scan. After measuring fasting blood glucose, the patients were intravenously injected with ^18^F-FDG (mean dose was 4.51 ± 0.77 MBq/kg), and placed in a quiet, warm and dark place to rest for 45~ 60 min. After urination, the patients were subjected to PET/CT scan. CT scan used the CareDose 4D technology (the tube current during CT scan is automatically adjusted according to patient’s body size, anatomy and tissue density), with tube voltage 100 kV, screw pitch 0.8, rotation time of bulb tube monolayer 0.5 s, layer thickness 5 mm, and reference milliampere seconds 60~ 180 mAs. PET scan was conducted immediately after CT scan with 3D mode. The scanning area ranged from skull base to upper femur, and acquisition time was 2 min/bed. Syngo TureD system was used to reconstruct images, forming cross-sectional, coronal and sagittal tomographic images, and three-dimensional projection images.

### CT diagnosis of nonalcoholic fatty liver disease (NAFLD)

The diagnosis of NAFLD was only based on CT images. Four regions of interest with a diameter of 4 cm were drawn on the left liver lobe, right anterior liver lobe, upper segment and lower segment of right posterior liver lobe, respectively. Two regions of interest with a diameter of 2 cm were drawn on the spleen (the two ROIs were 1.5 cm apart in depth) [12]. The average CT values of liver and spleen were calculated. If the CT value _spleen_/CT value _liver_ was greater than 1.1, and the subject had no prior history of alcohol consumption, then this subject was diagnosed as NAFLD [13]. The selected ROIs did not include biliary structures, major arterial and venous vessels, and the area of the hepatic cyst.

### Calculation of abdominal fat volume

Abdominal fat volume was divided into visceral adipose tissue (VAT) volume and subcutaneous adipose tissue (SAT) volume. The body CT image was reconstructed to 5 mm thickness. By using CT volume quantification software, VAT and SAT were sketched layer by layer from the S1 vertebra body up to abdominal muscle (25 continuous layers, 125 mm in total) [14]. Fat was defined as any voxel between − 190 to − 60 HU. VAT and SAT volumes were automatically calculated and recorded.

### Quantitative analysis of myocardial glucose uptake

**Fig. 2:**
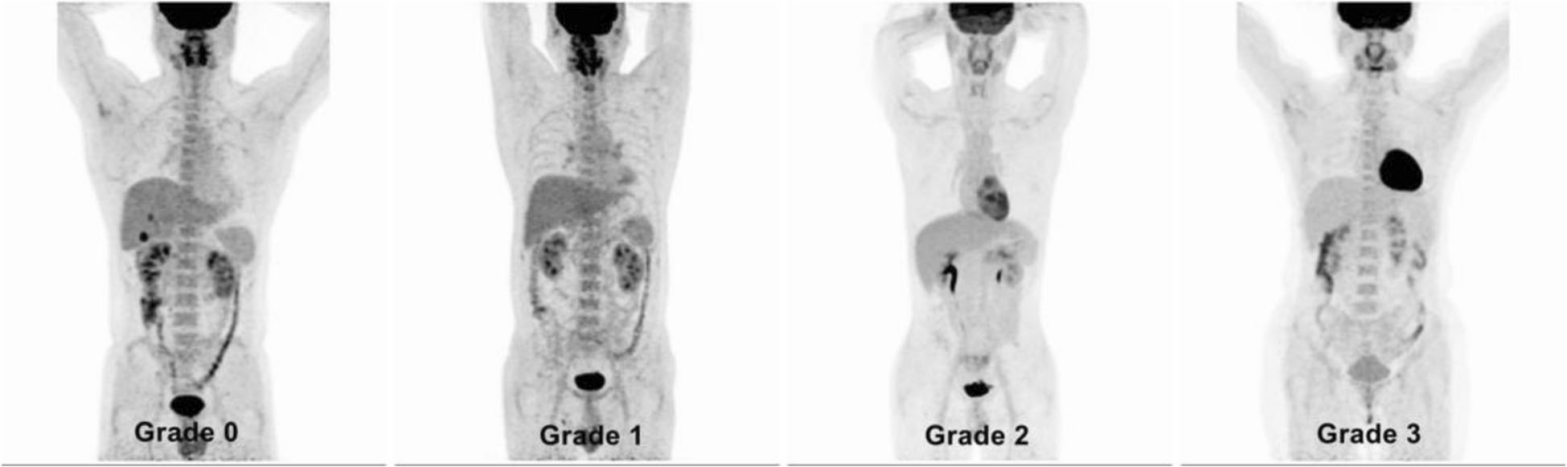
Visual grading scale of myocardial FDG uptake. (^18^F-FDG PET/CT maximal intensity projection images)

First, two experienced nuclear medicine physicians were asked to categorize myocardial glucose uptake into four grades based on visual evaluation [15]: grade 0 = minimal uptake, grade 1 = mostly minimal or mild uptake, grade 2 = mostly intense or moderate uptake, and grade 3 = homogeneously intense uptake (Fig. 2). When the grading was inconsistent between the two physicians, it was determined by mutual consultation. Next, the regions of interest on left ventricle were sketched layer by layer on the PET/CT combined images, and Siemens TrueD software was used to automatically calculate the averaged ^18^F-FDG standard uptake of the entire left ventricle (SUVmean). When the subject had low myocardial uptake as a blood pool, an ROI consisting of double U-shaped lines with about 1 cm distance was drawn along the border of the left ventricle on the transaxial PET/CT images [16, 17].

### Statistical analysis

SPSS 23.0 statistical software was used for statistical analysis. The measurement data were first tested by Kolmogorov-Smirnov test to check its normality. The normally distributed data was expressed as mean ± standard deviation, and the non-normally distributed data was expressed as median (P25, P75). One-way ANOVA was used to compare the mean values among multiple groups with normal distribution, and Kruskal-Wallis test was used to compare the median of non-normally distributed groups. The counting data was expressed in terms of frequency and percentage. χ^2^ test was used to compare ratios between groups. When any theoretical frequencies in the list were less than 1, the Fisher exact test was conducted. ROC curve analysis was used to assess the efficacy of using visual grading of myocardial glucose uptake to predict diabetes. Spearman correlation analysis was used to analyze the correlations between visual grading of myocardial glucose uptake and its possible influencing factors. The independent influencing factors of myocardial glucose uptake grade were analyzed by multivariate Logistic regression, and the regression coefficients, Odds Ratio (OR) and 95% confidence intervals were calculated. *P* values were calculated by two-sided test. *P* < 0.05 was considered statistically significant.

## Results

### Baseline characteristics of study subjects

A total of 191 subjects were included in the study, with an averaged age of 48.8 ± 9.0 years old. 67.5% (129/191) of the subjects were male, with the mean BMI of 24.8 ± 2.9 kg/m^2^. Among them, 40.8% (78/191) were smokers, 36.6% (70/191) were drinkers, 13.6% (26/191) had impaired fasting glucose, 13.1% (25/191) had diabetes, and 17.3% (30/191) had NAFLD. The characteristics of NFG, IFG and DM groups, as well as the comparisons are shown in Table 1. Compared to NFG and IFG groups, DM group showed significant cardiovascular-related metabolic disorders, including overweight/obesity (significant increase in BMI and VAT), high NAFLD proportion, abnormal liver functions (elevated ALT and γ-GT levels), abnormal glucose metabolism (increased fasting blood glucose, HbA1c and fasting insulin), abnormal lipid metabolism (elevated TC, TG, LDL-C, and TC/HDL-C levels, decreased HDL-C level), and insulin resistance (increased HOMA-IR) (*P* < 0.05 for all).

**Table 1:** Baseline characteristics of study subjects

Parameters	NFG (*n* = 140)	IFG (*n* = 26)	Diabetes (*n* = 25)	*P* value
Male (%)	65.0	65.4	84.0	0.169
Age (years)	48.0 ± 9.0	51.4 ± 9.1	50.2 ± 8.3	0.143
BMI (kg/m^2^)	24.6 ± 2.9	24.5 ± 2.2	26.6 ± 3.2	0.004^*^
Current smokers (%)	37.9	42.3	56.0	0.233
Current drinkers (%)	35.7	34.6	44.0	0.711
ALT (u/L)	24.0 (16.3, 32.0)	25.5 (19.0, 36.0)	28.9 (21.5, 54.5)	0.020^*#^
AST (u/L)	18.9 ± 4.7	19.7 ± 6.7	21.3 ± 8.1	0.128
γ-GT (u/L)	27.0 (19.0, 47.8)	36.0 (20.5, 70.8)	46.0 (32.0, 90.0)	< 0.001^*#^
TC (mmol/ L)	4.95 ± 0.95	5.11 ± 1.09	5.59 ± 1.28	0.015^*^
TG (mmol/ L)	2.16 (1.52, 2.96)	2.51 (1.87, 3.67)	3.20 (2.39, 5.83)	0.001^*#^
HDL-C (mmol/ L)	1.19 ± 0.27	1.23 ± 0.33	0.99 ± 0.19	0.002^*^
LDL-C (mmol/ L)	2.44 ± 0.60	2.43 ± 0.56	2.86 ± 0.83	0.009^*^
TC/HDL-C	4.36 ± 1.29	4.41 ± 1.39	5.80 ± 1.58	< 0.001^*^
Lipoprotein (a) (mg/L)	63.0 (44.0, 115.0)	87.0 (44.0, 151.8)	66.0 (42.5, 126.5)	0.518^#^
TSH (μIU/ml)	2.09 (1.51, 3.23)	2.03 (1.22, 2.60)	2.33 (1.58, 2.72)	0.454^#^
Fasting glucose (mmol/L)	5.31 ± 0.42	6.40 ± 0.19	8.18 ± 1.61	< 0.001^*^
HbA1c (%)	5.35 ± 0.35	5.76 ± 0.50	7.04 ± 1.35	< 0.001^*^
Fasting insulin (mIU/L)	5.98 (4.44, 8.11)	5.63 (5.10, 7.99)	9.13 (5.68, 13.24)	0.001^*#^
HOMA-IR	1.39 (1.01, 1.98)	1.56 (1.42, 2.31)	3.33 (2.42, 4.68)	< 0.001^*#^
NAFLD (%)	14.3	15.4	36.0	0.029^*^
VAT volume (cm^3^)	1281 (875, 1709)	1452 (1114, 1714)	1699 (1364, 2068)	0.002^*#^
SAT volume (cm^3^)	1778 (1491, 2217)	1736 (1330, 2343)	1714 (1452, 2277)	0.999^#^

*BMI:* body mass index, *ALT*: glutamic-pyruvic transaminase, *AST*: glutamic-oxalacetic transaminease*, γ-GT*: glutamyl transpeptidase, *TC*: total cholesterol, *TG*: triglyceride,*HDL-C*: high density lipoprotein cholesterol, *LDL-C*: low density lipoprotein cholesterol, *NAFLD*: nonalcoholic fatty liver disease, *VAT:* visceral adipose tissue, *SAT*: subcutaneous adipose tissue

HOMA-IR = fasting glucose×fasting insulin /22.5

* indicates statistically siegnificant difference, # indicates Kruskal-Wallis test.

### Correlation between myocardial glucose uptake and diabetes

**Fig. 3:**
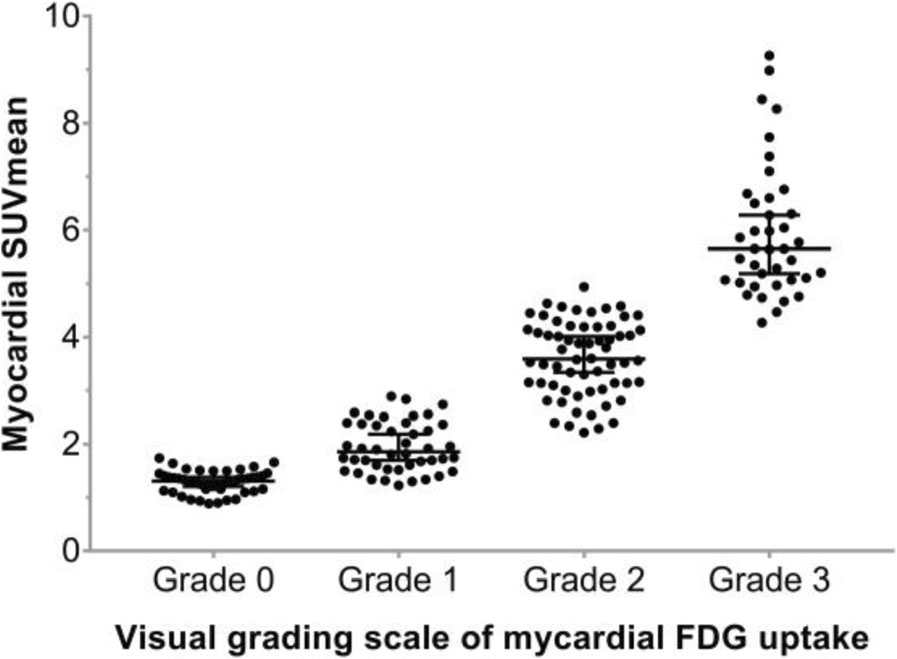
Myocardial SUVmean for each visual grade. SUVmean of grade 0–3 is 1.29 ± 0.21, 1.95 ± 0.47, 3.60 ± 0.70, 5.97 ± 1.25, respectively. (F = 338.14, *P* < 0.001)

As shown in Fig. 2, the myocardial FDG uptake was divided into 4 grades based on visual evaluation. Among the 191 subjects, grade 0, 1, 2, 3 accounted for 24.1%, 23.0%, 32.5% and 20.4% of the patients, respectively, and the corresponding SUVmean were 1.29 ± 0.21, 1.95 ± 0.47, 3.60 ± 0.70 and 5.97 ± 1.25, which were significantly different among all groups (F = 338.14, *P* < 0.001, Fig. 3). The visual grading of myocardial FDG uptake was positively correlated with SUVmean (*r* = 0.941, *P* < 0.001).

**Fig. 4:**
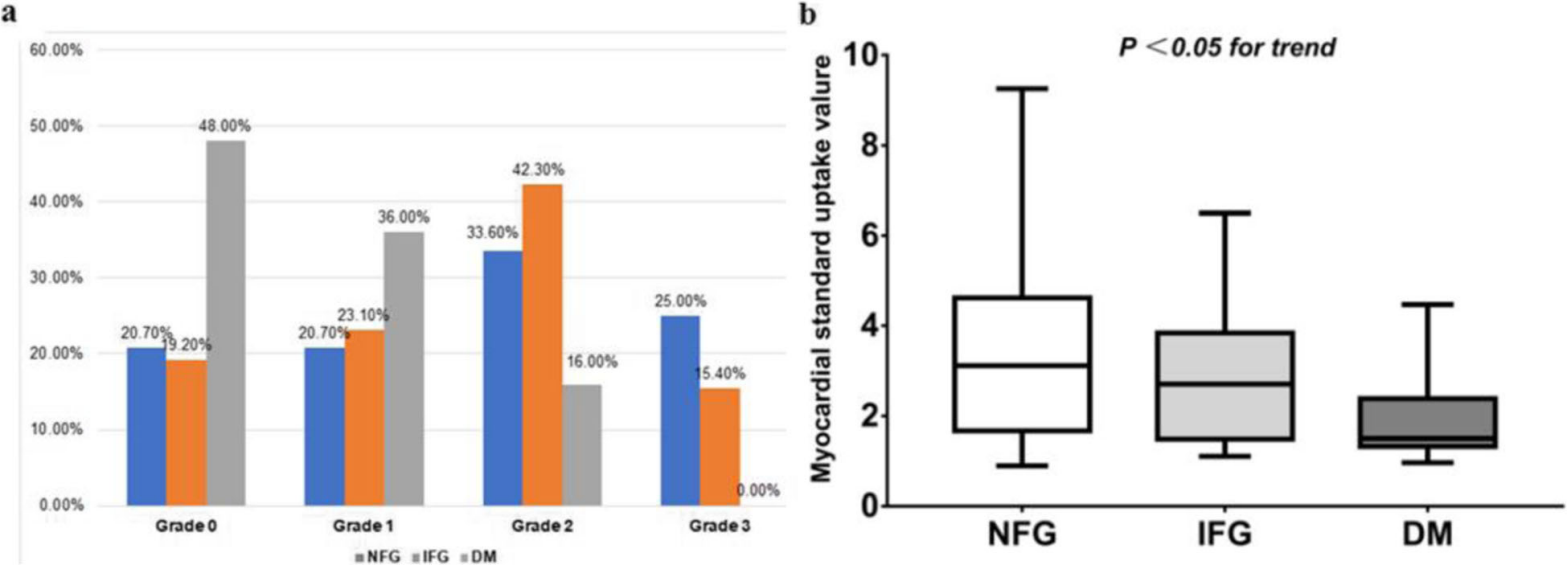
Correlation between myocardial glucose uptake and diabetes. **a**, the distribution of NFG, IFG and DM groups in each myocardial glucose uptake grade; **b**, the differences in SUVmean between NFG, IFG and DM groups (NFG: 3.42 ± 1.92, IFG: 2.86 ± 1.56, DM: 1.94 ± 0.96, *P* < 0.05)

**Fig. 5:**
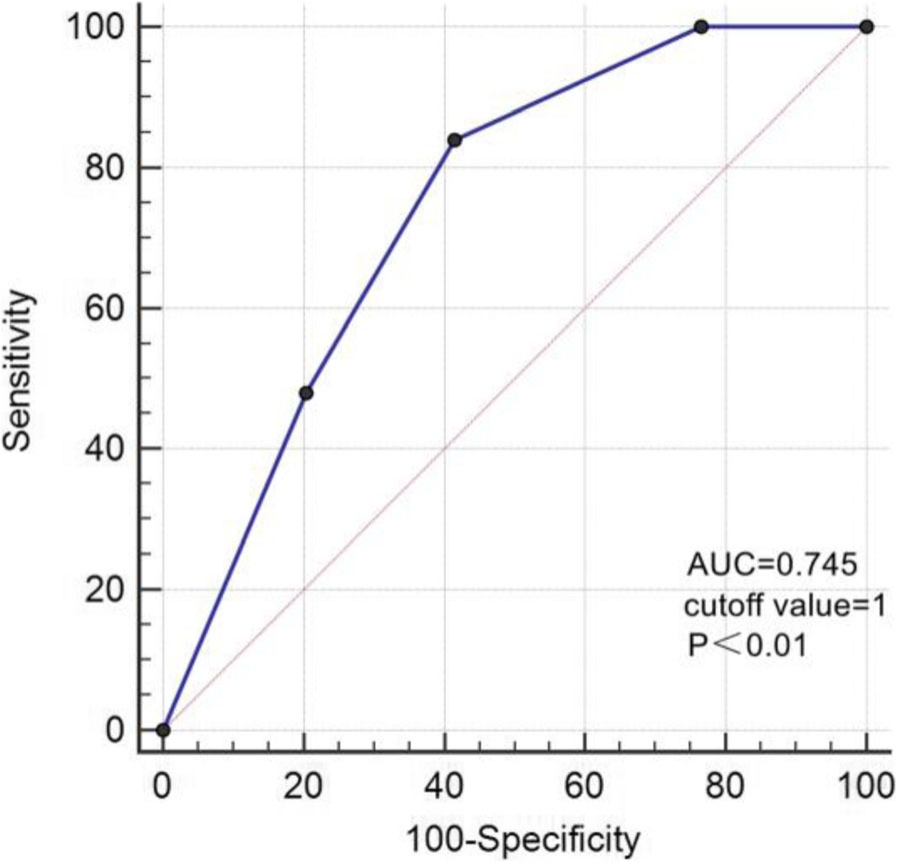
ROC curve analysis was used to assess the efficacy of using visual grading of myocardial FDG uptake to predict diabetes. When FDG uptake ≤ Grade 1, the sensitivity of predicting diabetes was 84%, the specificity was 58.43%, AUC = 0.745, and 95% confidence interval was 0.677 ~ 0.805 (z = 5.859, *P* < 0.001)

The SUVmean of NFG, IFG and DM groups were 3.42 ± 1.92, 2.86 ± 1.56 and 1.94 ± 0.96, respectively, significantly different among the three groups (F = 7.69, *P* = 0.01, Fig. 4b). Comparisons between any two groups showed that, the SUVmean of DM group was significantly lower than IFG and NFG groups (*P* < 0.05); the SUVmean of IFG group was lower than NFG group, but not significantly (*P* = 0.312). Moreover, in the DM group, up to 48.0% showed myocardial FDG uptake in grade 0, 36.0% were in grade 1, and none of them was in grade 3 (Fig. 4a). The ROC curve analysis of myocardial FDG uptake was used to predict the incidence of diabetes. When myocardial FDG uptake ≤ Grade 1, the prediction sensitivity was 84.0%, and the specificity was 58.4%, with AUC of 0.745, and 95% confidence interval of 0.677 ~ 0.805 (z = 5.859, *P* < 0.001, Fig. 5).

### The effects of cardiovascular-related metabolic disorders on myocardial FDG uptake

The correlations between visual grading of FDG uptake and the subjects’ general characteristics, as well as their metabolic parameters were analyzed by univariate analysis (Table 2). The analysis showed that myocardial FDG uptake had significant positive correlation with HDL-C, and significant negative correlations with BMI, smoking, drinking, ALT, γ-GT, TG, TC/HDL-C, fasting blood glucose, HbA1c, fasting insulin, HOMA-IR, NAFLD ratio, and VAT volume. The correlation coefficients are shown in Table 2. According to the ROC curve, grade 0 and grade 1 were defined as poor FDG uptake, and grade 2 and grade 3 were defined as good FDG uptake. Based on these two categories, the multivariate Logistic stepwise regression analysis showed that gender (male), HOMA-IR and NAFLD were independent risk factors for poor myocardial FDG uptake (OR > 1, *P* < 0.01, Table 3).

**Table 2:** Correlation between visual grading of myocardial FDG uptake and metabolic factors

Parameters	Grade 0 (*n* = 46)	Grade 1 (*n* = 44)	Grade 2 (*n* = 62)	Grade 3 (*n* = 39)	r	*P* value
Male (%)	89.1	72.7	61.3	46.2	− 0.319	< 0.001^*^
Age (years)	48.0 ± 7.9	50.7 ± 9.7	49.8 ± 9.2	48.8 ± 9.0	0.107	0.139
BMI (kg/m^2^)	25.6 ± 3.1	25.3 ± 2.9	24.3 ± 2.7	24.1 ± 2.9	−0.176	0.015^*^
Current smokers (%)	34.6	24.4	26.9	14.1	−0.222	0.002^*^
Current drinkers (%)	27.1	31.4	28.6	12.9	−0.161	0.026^*^
ALT (u/L)	30.5 (19.8, 42.0)	25.5 (21.0, 35.8)	22.0 (18.0, 30.3)	19.0 (14.0, 29.0)	−0.281	< 0.001^*^
AST (u/L)	20.8 ± 7.0	19.6 ± 6.0	18.8 ± 4.4	18.1 ± 4.6	−0.127	0.080
γ-GT (u/L)	44.0 (27.8, 72.8)	34.0 (23.3, 68.5)	25.0 (18.0, 43.8)	24.0 (15.0, 37.0)	−0.345	< 0.001^*^
TC (mmol/ L)	5.20 ± 1.36	5.10 ± 0.85	4.84 ± 0.81	5.17 ± 1.09	−0.026	0.719
TG (mmol/ L)	2.78 (1.77, 4.01)	2.53 (1.78, 3.81)	2.11 (1.45, 2.67)	2.03 (1.52, 2.71)	−0.231	0.001^*^
HDL-C (mmol/ L)	1.05 ± 0.27	1.09 ± 0.20	1.24 ± 0.28	1.27 ± 0.30	0.326	< 0.001^*^
LDL-C (mmol/ L)	2.60 ± 0.84	2.53 ± 0.54	2.36 ± 0.51	2.55 ± 0.67	−0.042	0.569
TC/HDL-C	5.21 ± 1.79	4.81 ± 1.19	4.09 ± 1.16	4.22 ± 1.24	−0.291	< 0.001^*^
Lipoprotein(a) (mg/L)	61.5 (37.8, 131.0)	64.0 (49.0, 125.0)	65.5 (44.8, 116.5)	73.0 (37.0, 122.0)	−0.006	0.934
TSH (μIU/ml)	2.02 (1.44, 2.83)	2.03 (1.47, 2.64)	2.16 (1.56, 3.53)	2.16 (1.48, 3.25)	0.093	0.201
Fasting glucose (mmol/ L)	6.28 ± 1.57	5.97 ± 1.16	5.64 ± 1.10	5.45 ± 0.51	−0.219	0.002^*^
HbA1c(%)	5.85 ± 1.01	5.81 ± 0.98	5.45 ± 0.69	5.45 ± 0.44	−0.173	0.018^*^
Fasting insulin (mIU/L)	7.90 (5.46, 10.40)	7.40 (4.86, 9.92)	5.70 (4.51, 6.78)	5.20 (4.00, 6.89)	−0.322	< 0.001^*^
HOMA-IR	2.17 (1.38, 2.88)	1.91 (1.36, 2.47)	1.29 (1.06, 1.63)	1.37 (0.95, 1.60)	−0.365	< 0.001^*^
NAFLD (%)	54.5	24.2	18.2	3.0	−0.337	< 0.001^*^
VAT volume (cm^3^)	1462 (1214, 1833)	1571 (1042, 1900)	1339 (950, 1731)	1084 (766, 1450)	−0.239	0.001^*^
SAT volume (cm^3^)	1693 (1354, 2021)	1712 (1396, 2190)	1781 (1516, 2183)	1841 (1490, 2375)	0.116	0.109

*BMI*: body mass index, *ALT*: glutamic-pyruvic transaminase*, AST*: glutamic-oxalacetic transaminease, *γ-GT*: glutamyl transpeptidase, *TC:* total cholesterol, *TG*: triglyceride,HDL-C: high density lipoprotein cholesterol, *LDL-C*: low density lipoprotein cholesterol, *NAFLD*: nonalcoholic fatty liver disease, *VAT*: visceral adipose tissue, *SAT*: subcutaneous adipose tissue

HOMA-IR = fasting glucose×fasting insulin /22.5

* indicates statistically significant difference.

**Table 3:** Logistic regression analysis of factors affecting myocardial FDG uptake

Variables	Regression coefficient	OR	95% CI	*P* value
Male	1.045	2.844	1.390~ 5.816	0.004
HOMA-IR	0.562	1.755	1.207~ 2.511	0.003
NAFLD	1.126	3.082	1.171~ 8.111	0.023

*NAFLD*: nonalcoholic fatty liver disease

HOMA-IR = fasting glucose×fasting insulin /22.5

## Discussion

In this study, we found that visual grading of myocardial FDG uptake had a very good positive correlation with myocardial SUVmean (*r* = 0.941, *P* < 0.001), thereby it can reflect the levels of fasting myocardial glucose metabolism. Under physiological conditions, FFA and glucose oxidation are the major sources of myocardial energy. The process of substrate selection is dynamic and largely depends on substrate availability, oxygen concentration, and myocardial workload [1]. Studies have shown that, in static whole-body PET/CT scan, the myocardial glucose usage during fasting is largely variable, which indirectly reflects the flexibility of myocardial substrate utilization [15, 18]. Among the 191 subjects in our study, the myocardial SUVmean ranged from 0.89 to 9.26, and subjects with visual grade 0–3 accounted for 24.1%, 23.0%, 32.5% and 20.4%, respectively. These results were consistent with the previous studies.

Myocardial energy metabolism disorders in diabetes is an important cause of CAD and heart failure [1, 5, 19]. Long-term abnormal glucose and lipid metabolisms, as well as insulin resistance, can lead to cardiac microvascular endothelial cell proliferation, basement membrane thickening, oxygen utilization reduction, degeneration of heart myofibers and perivascular fibers, and deposition of intracoronary and intramyocardial glycoproteins, collagen fibers, triglycerides and cholesterol, all of which can result in coronary artery lumen stenosis, myocardial energy metabolism disorders, decreased systolic/diastolic function, and eventually lead to CAD and heart failure. In this study, we found that DM group had multiple cardiovascular metabolic disorders, such as overweight/obesity, abnormal glucose and lipid metabolisms, NAFLD, and insulin resistance. Moreover, up to 84% of DM group showed poor FDG uptake (Grade 0/1), decreased variation range of SUVmean, and significantly lower myocardial SUVmean, as compared to IFG and NFG groups (Fig. 4b). These results indicate that in diabetes, the myocardial glucose metabolism and the flexibility of substrate utilization are both reduced.

When using myocardial FDG uptake less or equal to grade 1 to predict diabetes, the sensitivity was 84.0%, but the specificity was only 58.4%, suggesting that diabetes is not the only factor that leads to reduced myocardial glucose metabolism. Furthermore, the univariate analysis indicates that multiple metabolic factors can cause decreased myocardial glucose metabolism, which is consistent with the study from Kim G et al. [20]. However, their study used the ratio of heart SUV to liver SUV to reflect the myocardial FDG uptake, and it is still questionable that whether these two SUV values are collinear in insulin resistance (in our preliminary study, we found a weak correlation between heart SUVmean and liver SUVmean, *r* = − 0.151, *P* = 0.037); meanwhile, their study did not describe the percentage of NAFLD patients in study population, but we found NAFLD, gender (male), HOMA-IR are independent risk factors for poor myocardial FDG uptake.

Steady-state insulin assessment model (HOMA-IR) can reflect the degree of whole body insulin resistance during fasting. Since myocardium is one of the insulin target organs, when myocardium is insulin resistant, myocardial FDG uptake will decrease. Our study shows that HOMA-IR is an independent risk factor for poor myocardial FDG uptake (OR = 1.755, *P* = 0.003). It has been shown [21] that the major factors contributing to cardiac insulin resistance are oxidative stress, hyperglycemia, hyperlipidemia, dysregulated secretion of adipokines/cytokines, and inappropriate activation of renin-angiotensin II-aldosterone system (RAAS) and sympathetic nervous system. When glucose oxidation is decreased in cardiomyocytes, the fatty acids uptake and lipid metabolism are enhanced, which stimulates the tricarboxylic acid (TCA) cycle. The increased TCA cycle elevates citric acid levels, inhibits phosphofructokinase activity, reduces glycolysis rate, and activates the phosphorylation of pyruvate dehydrogenase, which then leads to inhibited pyruvate complex. All of these changes can result in increased acidosis in cardiomyocytes, sarcoplasmic reticulum swelling, and Ca^2^ influx blockage. During acidosis, H^+^ and Ca^2+^ competitively bind to myosin, causing the failure of excitement-contraction coupling and eventually leading to reduced myocardial contractility [22].

When the volume of fat tissue, which is responsible for energy storage, is saturated, excessive fat is ectopically deposited in liver, causing lipotoxicity and insulin resistance [23]. NAFLD is the manifestation of metabolic syndrome in liver, and is also an important risk factor for cardiovascular disease. Recent studies have reported that NAFLD is associated with altered high-energy phosphate metabolism [24], and subclinical myocardial remodeling and dysfunction [25]. Interestingly, in our study, we found NAFLD was also an independent risk factor for poor FDG uptake in myocardium (OR = 3.082, *P* = 0.023), suggesting that NAFLD is also involved in the energy substrate metabolism of myocardium. Several large population-based cohort studies have demonstrated that moderately elevated GGT levels, which are potential markers for NAFLD and atherosclerosis, are independently associated with increased risk of heart failure [26]. Therefore, our study provides further evidence to demonstrate the possible link between NAFLD and heart failure.

The current study also exhibits several limitations. Since this study was a retrospective study, none of the subjects underwent oral glucose tolerance test. Thereby some subjects in the NFG group might have impaired glucose tolerance (IGT), which may affect the differences between IFG and NFG group (IFG and IGT both belong to pre-diabetes). In addition, the study subjects were free of cardiovascular disease, and the diabetes group only accounted for 13.1%. Although the diabetes ratio was consistent with the diabetes incidence in local population [27], the number was relatively small, thereby we couldn’t investigate the influencing factors of myocardial glucose metabolism in diabetic patients.

## Conclusions

In summary, static whole-body PET/CT is a simple and easy method to study myocardial glucose metabolism. The visual grading of myocardial FDG uptake had highly positive correlation with myocardial SUVmean. Diabetes is significantly associated with cardiovascular-related metabolic disorders and reduced myocardial glucose metabolism. Using decreased myocardial glucose metabolism to predict diabetes is highly sensitive, but with a relatively low specificity (only 58.4%), suggesting that diabetes is not the only factor causing reduced myocardial glucose metabolism. Univariate analysis demonstrated that multiple factors (gender, overweight/obesity, lifestyle, laboratory parameters, and NAFLD) could lead to decreased myocardial glucose metabolism. Moreover, multivariate regression analysis showed that gender (male), HOMA-IR and NAFLD were independent risk factors for poor myocardial FDG uptake.

## Abbreviations

DM: Diabetes mellitus
FDG: Fluorodeoxyglucose
HDL: High density lipoprotein
HF: Heart failure
LDL: Low density lipoprotein
MBq: Megabecquerel
NAFLD: Nonalcoholic fatty liver disease
PET: Positron emission tomography
ROI: Region of interest
TC: Total cholesterol
TG: Triglycerides
